# Common Brain Substrates Underlying Auditory Speech Priming and Perceived Spatial Separation

**DOI:** 10.3389/fnins.2021.664985

**Published:** 2021-06-17

**Authors:** Junxian Wang, Jing Chen, Xiaodong Yang, Lei Liu, Chao Wu, Lingxi Lu, Liang Li, Yanhong Wu

**Affiliations:** ^1^School of Psychological and Cognitive Sciences and Beijing Key Laboratory of Behavior and Mental Health, Peking University, Beijing, China; ^2^Department of Machine Intelligence, Peking University, Beijing, China; ^3^Speech and Hearing Research Center, Key Laboratory on Machine Perception (Ministry of Education), Peking University, Beijing, China; ^4^School of Nursing, Peking University, Beijing, China; ^5^Center for the Cognitive Science of Language, Beijing Language and Culture University, Beijing, China; ^6^Beijing Institute for Brain Disorders, Beijing, China

**Keywords:** auditory speech priming, perceived spatial separation, speech recognition, speech motor system, common brain substrate, unmasking

## Abstract

Under a “cocktail party” environment, listeners can utilize prior knowledge of the content and voice of the target speech [i.e., auditory speech priming (ASP)] and perceived spatial separation to improve recognition of the target speech among masking speech. Previous studies suggest that these two unmasking cues are not processed independently. However, it is unclear whether the unmasking effects of these two cues are supported by common neural bases. In the current study, we aimed to first confirm that ASP and perceived spatial separation contribute to the improvement of speech recognition interactively in a multitalker condition and further investigate whether there exist intersectant brain substrates underlying both unmasking effects, by introducing these two unmasking cues in a unified paradigm and using functional magnetic resonance imaging. The results showed that neural activations by the unmasking effects of ASP and perceived separation partly overlapped in brain areas: the left pars triangularis (TriIFG) and orbitalis of the inferior frontal gyrus, left inferior parietal lobule, left supramarginal gyrus, and bilateral putamen, all of which are involved in the sensorimotor integration and the speech production. The activations of the left TriIFG were correlated with behavioral improvements caused by ASP and perceived separation. Meanwhile, ASP and perceived separation also enhanced the functional connectivity between the left IFG and brain areas related to the suppression of distractive speech signals: the anterior cingulate cortex and the left middle frontal gyrus, respectively. Therefore, these findings suggest that the motor representation of speech is important for both the unmasking effects of ASP and perceived separation and highlight the critical role of the left IFG in these unmasking effects in “cocktail party” environments.

## Introduction

How do our brains deal with a complex scene where listeners need to selectively detect, follow, and recognize a speaker’s words (target) when multiple people are talking (masker) at the same time (i.e., the “cocktail party” problem) (Cherry, 1953; Schneider et al., 2007; McDermott, 2009; Bronkhorst, 2015)? Previous studies have shown that listeners can take advantage of diverse perceptual and/or cognitive cues, such as prior knowledge of the contents of the speech and/or the speaker’s voice (Freyman et al., 2004; Yang et al., 2007), information obtained from lip reading (Wu et al., 2017b), and perceived spatial separation (Freyman et al., 1999), to improve their recognition of target speech masked by non-target sounds (i.e., release from masking or unmasking). In real-life conditions, these unmasking cues are not alone, and several cues belonging to an auditory object influence the perception at the same time. However, a majority of relevant studies usually focused on the cognitive and neural mechanisms of a single one among these unmasking cues (Zheng et al., 2016; Wu et al., 2017a,b), and very few have investigated dual unmasking cues that are closer to a real-life condition (Du et al., 2011; Lu et al., 2018). Therefore, this study employed two unmasking cues in a unified paradigm to investigate neural bases across different unmasking effects.

Among multiple unmasking cues, the prior knowledge of the content and voice of the target speech [i.e., auditory speech priming (ASP)] and perceived spatial separation are two typical and effective cues. The ASP refers to a segment of the target phrase spoken by the target speaker, which is presented without interferences before a target–masker mixture, and it can improve recognition of the last keyword in the target speech, even though the last keyword does not appear in the segment (Freyman et al., 2004; Wu et al., 2012a,b, 2017a). The perceived spatial separation represents spatially separating sound images of target speech from those of competing speech, which can cause even larger unmasking effect on speech recognition (Freyman et al., 1999, 2004; Li et al., 2004; Bronkhorst, 2015). Studies of patients with schizophrenia suggest that ASP and perceived separation share some common features. Even though patients with schizophrenia exhibit deficiencies in speech perception and increased vulnerability to masking stimuli, they retain the ability of using ASP and perceived separation to improve their recognition of the target speech masked by two-talker speech (Wu et al., 2012, 2017a). Similarly, despite age-related declines in hearing, older adults can utilize ASP and perceived separation to improve their recognition of the targets under a noisy environment just as well as do younger adults (Li et al., 2004; Ezzatian et al., 2011). These studies propose that certain top-down auditory mechanisms underlying the unmasking effects of ASP and perceived separation are preserved in patients with schizophrenia and older adults (Li et al., 2004; Ezzatian et al., 2011; Wu et al., 2012, 2017a). Researchers have claimed that the ASP helps listeners maintain the target’s voice and content in working memory and may facilitate grouping the target speech to enhance the listener’s selective attention to the target (Freyman et al., 2004; Schneider et al., 2007; McDermott, 2009; Ezzatian et al., 2011; Wu et al., 2012a,b; Carlile, 2015; Wang et al., 2019). The perceived separation mainly works via the head-shadowing effect increasing the signal-to-masker ratio (SMR) in the ear close to the target, and/or the neurophysiological effect of disparity in interaural time between targets and maskers, both of which enhance selective attention to the target speech (Freyman et al., 1999; Li et al., 2004). Accordingly, the unmasking effects of ASP and perceived separation may both contribute to the high-level processing, such as enhanced selective attention to the target speech. Moreover, the selective attention might allocate more cognitive resources to the motor representation of speech that is beneficial for speech recognition under “cocktail party” listening conditions (Wu et al., 2014). The intersecting processing of the unmasking effects induced by ASP and perceived separation is predicted to occur in the high-level processing.

Previous studies have no consensus on whether two unmasking cues are processed independently or interdependently. If an additive effect was observed, i.e., the combined effect of two cues is equivalent to the sum of their individual effects, researchers conclude that these two cues are processed independently in separate brain regions (Bronkhorst, 2015). Otherwise, the non-additive effect indicates intersecting processing of these cues in overlapping brain regions. Studies have shown varied additives of two cues; for instance, Du et al. (2011) found an additive effect of the differences in fundamental frequency and spatial location during speech segregation. Lu et al. (2018) found an additive effect of emotional learning (of the target voice) and perceived separation on improving speech recognition. Despite this, Darwin et al. (2003) found that the effect of the differences in fundamental frequency and vocal-tract length is larger than the sum of their individual effects during speech recognition. Freyman et al. (2004) found that the benefit of ASP was more significant when the target and maskers were perceived to be co-located than when they were perceived to be separated, indicating the non-additive effect of ASP and perceived separation. These suggest that the combinations of different cues result in varied additive effects. In the case of ASP and perceived separation, their benefits to speech recognition cannot be added, suggesting the existence of intersections of their neural underpinnings. The present study aims to investigate how ASP and perceived separation improve the recognition of the target speech in a complex scene and reveal the neural bases underlying their unmasking effects.

Despite a lack of studies on neural mechanisms underlying dual unmasking effects, some researchers have separately investigated neural bases of the unmasking effects of ASP and perceived spatial separation. They have found that ASP and perceived separation mainly activated the ventral and dorsal pathways in the auditory system, respectively. Notably, both of them activate the left inferior frontal gyrus (IFG) (Zheng et al., 2016; Wu et al., 2017a). In addition, ASP and perceived separation both enhance the functional connectivity between the IFG and specific brain substrates, such as the left superior temporal gyrus and left posterior middle temporal gyrus for the ASP, and the superior parietal lobule for the perceived separation (Zheng et al., 2016; Wu et al., 2017a). This suggests that the speech motor system, especially the IFG (Du et al., 2014), may be the common brain area that is activated by both the unmasking effects of ASP and perceived separation. The left IFG is thought to be the speech-related area involved in both speech production and perception (Hickok and Poeppel, 2004; Liakakis et al., 2011). It exchanges information with other brain substrates located in the ventral and dorsal pathways in the auditory system (Friederici, 2012), and it is involved in unification operations during speech comprehension (Acheson and Hagoort, 2013). Moreover, the par triangularis of the IFG (TriIFG) is a junction in the IFG (Friederici, 2012; Frühholz and Grandjean, 2013). A dual stream model for auditory processing suggests that auditory information transferred via both ventral and dorsal streams terminates in the TriIFG (Frühholz and Grandjean, 2013). The TriIFG is thought to be the main locus of speech intelligibility in the IFG (Abrams et al., 2013), and it is especially important in the retrieval or selection of semantic information under adverse listening conditions (Skipper et al., 2007). The present study predicts that the speech motor system plays a critical role in the unmasking effects of ASP and perceived separation. In particular, the TriIFG is the interested brain substrate, and it is predicted to correlate with recognition accuracy of the target speech in a multitalker condition.

In summary, previous studies inconsistently suggest the existence of intersecting processing of dual unmasking cues. In the present study, we introduced ASP and perceived spatial separation in a “cocktail party” listening condition and combined them in a unified paradigm. We aim to first verify that the unmasking effects of ASP and perceived separation are not processed independently and further investigate the existence of their common neural bases, by using functional magnetic resonance imaging (fMRI). The existence of overlapping brain substrates is thought to be a primary investigation of the commonality of neural mechanisms underlying two unmasking effects. In the light of past results, we hypothesized that the unmasking effects of ASP and perceived separation intersect in the high-level processing, and the left IFG is the shared brain substrate critically involved in these unmasking effects.

## Materials and Methods

### Participants

Thirty-six participants (21 females, 15 males; mean age = 22.06 years, SD = 1.94 years; Min_*age*_ = 19 years, Max_*age*_ = 27 years) took part in the behavioral testing, and 27 of them (15 females, 12 males; mean age = 22 years, SD = 1.94 years; Min_*age*_ = 19 years, Max_*age*_ = 27 years) voluntarily participated in the follow-up fMRI testing. All participants were right-handed university students who spoke Mandarin. All of them had normal pure-tone hearing thresholds (≤25 dB HL) in each ear and had bilaterally symmetric hearing (≤15 dB HL). The hearing thresholds were measured by an audiometer (Aurical, 60645-1, Danmark) at frequencies of 125, 250, 500, 1,000, 2,000, 4,000, 6,000, and 8,000 Hz for 22 participants. The hearing thresholds of the remaining participants were measured by Apple iOS-based automated audiometry (Xing et al., 2016) at frequencies of 250, 500, 1,000, 2,000, 3,000, 4,000, 6,000, and 8,000 Hz in a sound booth. All participants gave their informed consent before the experiment and received a certain monetary compensation. The experimental procedures were approved by the Committee for Protecting Human and Animal Subjects in the School of Psychological and Cognitive Sciences, Peking University.

### Stimuli and Procedures

The mean duration of a sound stimulus was 3,711 ± 341 ms. Each speech stimulus comprised three parts: a priming stimulus [of the ASP condition or auditory non-speech priming (ANSP) condition], a target phrase, and a two-talker speech masker. It started with a priming stimulus, following which a target phrase mixed with a two-talker speech masker was presented. The speech stimuli were processed by head-related transfer functions (Qu et al., 2009) to simulate sounds from one of three azimuth angles (i.e., −90°, 0°, 90°) 30 cm from the center of the listeners’ heads in the horizontal plane. The target phrases and the two-talker speech masker were “non-sense” sentences in Chinese that were syntactically, but not semantically, correct. The target phrases, spoken by a young female (talker A), were three-word phrases. Each word of the target phrase contained two syllables. For example, one target phrase translated into English was “contest this employee” (keywords are underlined). The structure of these target phrases did not support any context for recognizing the keywords. There was no overlap in target phrases between behavioral and fMRI tests for each participant. In the fMRI testing, we used a minority of response trials as probes to monitor whether participants were doing a speech-recognition task. Target phrases of these response trials were modified by substituting the last keyword of the original target phrase with the first keyword of it, so that the first and the last keywords were identical.

Considering that the benefits of ASP and perceived separation are more significant when masked by the two-talker speech masker than other types of maskers (Freyman et al., 2004; Lu et al., 2018), we used the two-talker speech masker as the distractive sounds. The masker was a 47-s loop of a digitally combined continuous recording of Chinese non-sense sentences spoken by two young females (talkers B and C). No keyword in the speech masker appeared in target phrases. In the behavioral testing, the sound pressure level of the target phrases was fixed at 56 dBA SPL, and the sound levels of the maskers were adjusted to produce four SMRs: −12, −8, −4, and 0 dB. In the fMRI testing, the SMR was fixed at −4 dB (Wu et al., 2017a,b). All sound pressure levels were measured by an Audiometer Calibration and Electroacoustic Testing System (AUDit and System 824; Larson Davis, Provo, UT, United States). The SMRs were calibrated before applying the head-related transfer function.

**FIGURE 1:**
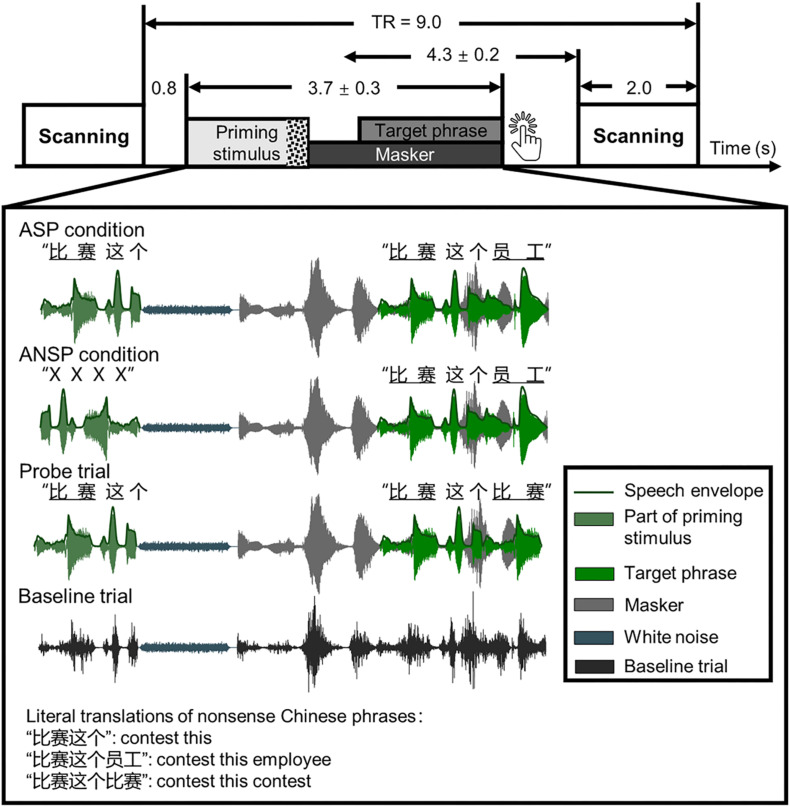
Schematic of a single trial presenting sound stimuli in a functional run. Sparse temporal sampling was used. Four kinds of sound structures of the functional runs are illustrated separately. The temporal midpoint of the sound stimulus was 4,300 ms prior to the onset of scanning for a given trial. The unit of time in this figure is second(s). TR, time to repeat.

The priming stimuli were manipulated differently in the ASP and the ANSP conditions (see the illustration in Figure 1). In the ASP condition, a priming stimulus was identical to a target phrase except that the last keyword of the target was replaced by a piece of white noise. The duration of the white noise matched that of the longest last keyword across all target phrases. The sound pressure level of the white noise was 10 dB lower than that of the corresponding target phrase (following Freyman et al., 2004) to ensure its perceived loudness being consistent. In the ANSP condition, a priming stimulus was produced as follows: The target segment containing the first two words of the target phrase was time reversed and connected to the same white noise as described above. The acoustic property (i.e., long-term spectrum, mean pitch) of this priming stimulus was close to that used in the ASP condition, but without semantic information.

Because the continuity and certainty of location help identify a target object and release it from masking (Kidd et al., 2005; Best et al., 2008), the perceived locations of the priming stimuli and the target phrases were fixed across conditions at the azimuthal 0° to prevent listeners from switching their attention along the spatial dimension. In the perceived separation condition, the masker was simulated from an azimuth of −90° or 90°. In the perceived co-location condition, all of the priming stimuli, target phrases, and maskers were simulated from the azimuthal 0°.

During the fMRI testing, we employed amplitude-modulated speech-spectrum noises (ASSNs) as the baseline condition. The ASSNs were obtained by (1) a fast Fourier transform algorithm returning the discrete Fourier transform of the original speech signals and then randomizing phases of all spectral components, (2) an inverse discrete Fourier transform to convert these modified Fourier outputs back into the time domain, (3) normalized amplitudes of these speech-spectrum noises, and (4) modulated speech-spectrum noises according to the amplitudes of the original speech signals extracted by a Hilbert transformation. As a result, the ASSN of the same length as the original speech had temporal fluctuations and spectra close to those of the speech stimuli, but sounded like noise.

### fMRI Testing

The fMRI testing featured two intraparticipant variables: (1) priming type (ASP, ANSP) and (2) perceived laterality relationship (separation, co-location). The combination of them led to four conditions: ASP and separation condition (i.e., the PS condition), ASP and co-location condition (i.e., the PC condition), ANSP and separation condition (i.e., the nPS condition), and ANSP and co-location condition (i.e., the nPC condition).

In functional runs, we used sparse temporal sampling to reduce the influence of machine noises (Hall et al., 1999; Zheng et al., 2016; Wu et al., 2017a,b). Consequently, the sounds were presented between scans. In each trial of the functional runs (see the illustration in Figure 1), a sound stimulus was presented 800 ms after the end of scanning of the previous trial so that the midpoint of presentation of the stimulus occurred approximately 4,300 ms before the onset of scanning for the given trial. This was done to ensure that the sound stimulus–evoked hemodynamic responses peaked during the scanning period (Wild et al., 2012a; Zheng et al., 2016; Wu et al., 2017a,b). A speech stimulus started with a priming stimulus that presented without interferences, following which a target phrase mixed with a two-talker speech was presented (the target phrase occurred 1,000 ± 200 ms after the onset of the masker, and they were terminated simultaneously). The participants were instructed to carefully listen to the sounds and press a key immediately when they determined that the first and the last keywords of the target phrase were identical (i.e., when they encountered response trials, they were required to make a key press).

The SMR was fixed at the −4 dB as the ASP was significantly advantageous in both the perceived separation and perceived co-location conditions in this SMR (see *Results*). We used the ASSN baseline condition as the controlling condition for four speech stimuli conditions, and the response trials as probes to monitor whether participants were doing a speech-recognition task. In each functional run, there were 10 trials for each of the five listening conditions (PS, PC, nPS, nPC, and baseline), six response trials, and 10 blank trials (no sound was presented between scans). These trials were randomly presented. A whole-course scanning for each participant consisted of an 8-minute structural scanning and four 10-minute functional runs. Before the formal testing, a training session was conducted outside the scanning room to ensure that participants understood their tasks in the fMRI testing. The stimuli were delivered via a visual and audio stimulation system for fMRI (Sinorad, Shenzhen, China), driven by MATLAB (MathWorks Inc., Natick, MA, United States). The sounds were bilaterally presented to listeners using a pair of MRI-compatible headphones (Sinorad, Shenzhen, China). Foam earplugs (Noise Reduction Rating: 29 dB; 3 M, 1100, Maplewood, United States) were provided to reduce scanner noises.

### fMRI Equipment

A 3.0-T Siemens Prisma MRI scanner (Siemens, Erlangen, Germany) was used to acquire blood oxygenation level–dependent gradient echo-planar images (spatial resolution: 112 × 112 × 62 matrix with a voxel size of 2 mm × 2 mm × 2 mm; acquisition time: 2,000 ms; time to repeat: 9,000 ms; echo time: 30 ms; flip angle: 90°; FoV: 224 mm × 224 mm). It provided high-resolution T1-weighted structural images (448 × 512 × 192 matrix with a voxel size of 0.5 mm × 0.5 mm × 1 mm; repetition time: 2,530 ms; echo time: 2.98 ms; flip angle: 7°).

### fMRI Data Processing and Analysis

#### Preprocessing

The fMRI data were analyzed by Statistical Parametric Mapping software (SPM12^1^). The preprocessing of the fMRI data consisted of the following steps: (1) the functional images were realigned to correct head movements. (2) T1-weighted structural images were coregistered with the mean realigned functional images. (3) T1-weighted images were segmented and normalized to the ICBM space template. (4) All functional images were warped by deformation parameters generated in the segmentation process. (5) All functional images were smoothed by a Gaussian kernel with 6-mm full width at half maximum. Because the repetition time was relatively long in such a sparse imaging paradigm, no slice timing was conducted in preprocessing (Wild et al., 2012a; Zheng et al., 2016; Wu et al., 2017a,b).

#### Random-Effects Analysis and *Post hoc* Tests

The first-level general linear model for each participant contained seven regressors in total: five for the listening conditions (PS, PC, nPS, nPC, and baseline), one for blank trials, and one for uninteresting/irrelevant response trials. Six realignment parameters of head movements were entered to account for residual movement-related effects. The blood oxygenation level–dependent response for each event was modeled using the canonical hemodynamic response function. Contrast images of “PS > baseline,” “PC > baseline,” “nPS > baseline,” and “nPC > baseline” for each participant, were entered into a 2 (priming type: ASP, ANSP) × 2 (perceived laterality relationship: separation, co-location) repeated-measure analysis of variance (ANOVA). Only clusters passing the cluster-level family wise error correction for multiple comparisons (we set at FWE corrected cluster-level threshold of *p*_*FWE*_ < 0.05) were entered into the *post hoc* tests. To identify the causes of main effects, *post hoc* paired-samples *t* tests were conducted by inclusively masking specific *t*-contrast images with the corresponding *F* contrasts (*p* < 0.001 at the voxel level, uncorrected). We calculated the ASP effect (i.e., “ASP > ANSP” contrast) by the formula (PS + PC) > (nPS + nPC) and the spatial unmasking effect (i.e., “perceived separation > perceived co-location” contrast) by the formula (PS + nPS) > (PC + nPC). Moreover, referring to the method in Wild et al. (2012a), we calculated the logical intersections of clusters activated by both the ASP effect and the spatial unmasking effect. Overlapping clusters containing more than 10 voxels were reported and used as regions of interest for behavioral–neural correlation analyses.

#### Correlation Analysis

To identify the correlation between brain activations and behavioral improvements by the ASP effect and the spatial unmasking effect, we conducted Spearman correlation analyses by using the IBM SPSS.20 software. As we have introduced in the *Introduction*, the left TriIFG is important in semantic processing of speech under adverse listening conditions. We used the left TriIFG that was activated by both unmasking effects as the region of interest in the correlation analyses and extracted contrast values within this overlapping cluster by the MarsBar toolbox (version 0.44^2^). The correlations for four contrasts (i.e., “PS > nPS,” “PC > nPC,” “PS > PC,” and “nPS > nPC”) were examined by using corresponding contrast values and behavioral improvements at the −4 dB SMR.

#### Psychophysiological Interaction Analysis

We conducted generalized form of context-dependent psychophysiological interaction (PPI) analyses (McLaren et al., 2012) to identify brain regions showing significant functional connectivity with the brain substrates of interest (i.e., the left IFG), by using the gPPI Toolbox (version 7.12^3^, based on SPM8). Each seed region was defined as a sphere with a 6-mm radius centered at the peak voxel. The first-level generalized PPI model contained all regressors in a first-level general linear model, additional PPI regressors (for the PS, PC, nPS, and nPC conditions, noise baseline trials, blank trials, and response trials), time courses of the seed region, and a constant (McLaren et al., 2012). Single-participant contrast images (“PS > nPS,” “PC > nPC,” “PS > PC,” and “nPS > nPC” contrasts) of the first-level generalized PPI model were subjected to second-level one-sample *t* tests to identify brain regions showing increased coactivation with the seed region due to corresponding contrasts. The level of significance was set at *p* < 0.001 uncorrected with an extent threshold with minimum cluster size of 20 voxels (Wandschneider et al., 2014).

### Behavioral Testing

Behavioral testing was conducted before the fMRI testing, and it featured three intraparticipant variables: (1) priming type (ASP, ANSP), (2) perceived laterality relationship (separation, co-location), and (3) SMR (−12, −8, −4, 0 dB). Variables for the priming type and perceived laterality relationship were the same as in the fMRI testing.

In each trial, a speech stimulus started with a priming stimulus, following which a target phrase mixed with a two-talker speech masker was presented. The onset of the target phrase was 1,000 ± 200 ms later than that of the masker, and they were terminated simultaneously. Participants started each trial by themselves, and they were instructed to verbally repeat the entire target phrase as much as possible when the sounds ended in each trial. The experimenter scored and calculated the number of correctly recognized syllables of the last keywords (participants were not aware that only the last keywords were scored). Each syllable of the last keyword was counted as one point. Before the formal testing, a training session was conducted to ensure that participants had understood the task of the behavioral testing.

Four combinations of priming types and perceived laterality relationships were assigned to four blocks, and their orders of presentation were counterbalanced across participants by using the Latin square order. The four SMRs were randomly ordered in each block. For each participant, 12 trials (12 target phrases) were conducted for each of 16 conditions. Sounds were binaurally presented to participants via the headphones (HD 650, Sennheiser electronic GmbH & Co., KG, Germany), driven by Presentation software (version 0.70).

## Results

### Behavioral Improvements Due to ASP and Perceived Spatial Separation

**FIGURE 2:**
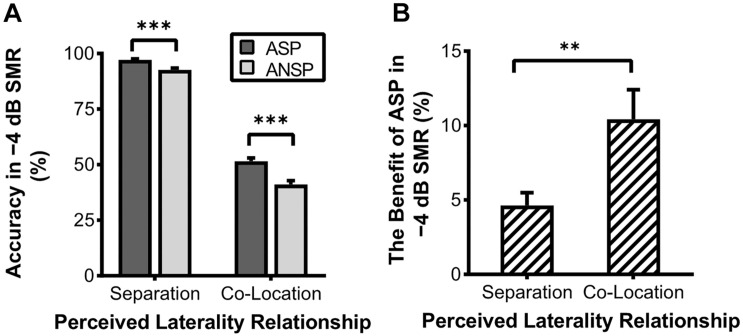
Accuracy of recognition of the last keyword in target phrases under the −4 dB SMR in the behavioral testing. **(A)** Comparisons of recognition accuracy under different priming types and perceived laterality relationships. **(B)** The enhanced recognition accuracy obtained through ASP against ANSP under different relationships of perceived laterality. The error bars represent standard errors. ^∗∗∗^*p* < 0.001, ^∗∗^*p* < 0.01.

In the behavioral testing, the 2 (priming type: ASP, ANSP) × 2 (perceived laterality relationship: separation, co-location) × 4 (SMR: −12, −8, −4, 0 dB) repeated-measured ANOVA showed significant main effects of priming type (*F*_1, 35_ = 34.98, *p* < 0.001, η_*p*_^2^ = 0.50), perceived laterality relationship (*F*_1, 35_ = 2,539.91, *p* < 0.001, η_*p*_^2^ = 0.99), and SMR (*F*_3, 105_ = 1,127.50, *p* < 0.001, η_*p*_^2^ = 0.97). Their interaction was also significant (*F*_3, 105_ = 7.33, *p* < 0.001, η_*p*_^2^ = 0.17). Simple-effect analyses (Bonferroni-corrected) showed that the ASP effect was not consistently significant between the two perceived laterality relationships (detailed results are provided in Supplementary Table 1). Only when the SMR was −4 dB did the ASP contrast to ANSP improve the recognition of the target under both the perceived separation (PS:0.97 ± 0.02, nPS:0.93 ± 0.05, *F*_1, 35_ = 29.05, *p* < 0.001) and the perceived co-location (PC:0.51 ± 0.09, nPC:0.41 ± 0.10, *F*_1, 35_ = 27.44, *p* < 0.001) conditions (see Figure 2A). In the −4 dB SMR, the benefits of ASP (recognition accuracy of ASP minus that of ANSP) between the two perceived laterality relationships were compared by paired-sample *t* tests. The results showed that the benefit of ASP under the perceived co-location condition (i.e., recognition accuracy of PC minus that of nPC; the benefit of ASP:0.10 ± 0.12) was greater than that under the perceived separation condition (i.e., recognition accuracy of PS minus nPS; the benefit of ASP:0.05 ± 0.05) (*t*_35_ = 3, *p* = 0.005, see Figure 2B). These results indicate that the unmasking effect of ASP decreases when the perceived spatial separation helps improve the recognition of the target speech.

### Brain Regions Activated by ASP and Perceived Spatial Separation

**FIGURE 3:**
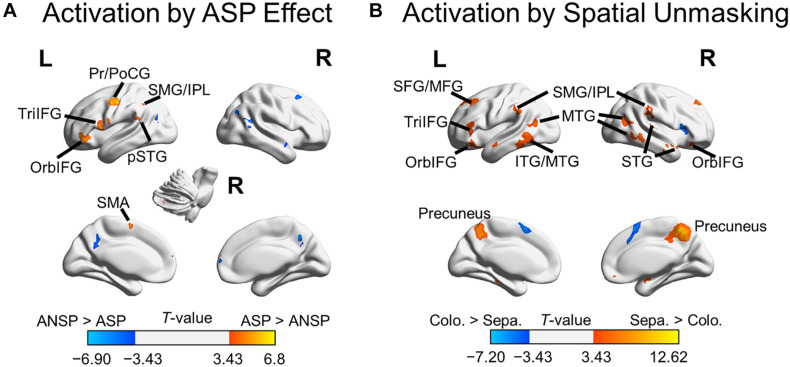
Areas of the brain activated by the ASP effect and spatial unmasking effect. **(A)** Clusters activated by the “ASP > ANSP” contrast (i.e., the ASP effect, the warm color) and by the “ANSP > ASP” contrast (the cold color). **(B)** Clusters activated by the “perceived separation > perceived co-location” contrast (i.e., spatial unmasking effect, the warm color) and by the “perceived co-location > perceived separation” contrast (the cold color). ITG, inferior temporal gyrus; MTG, middle temporal gyrus; PrCG, precentral gyrus; PoCG, postcentral gyrus; SFG, superior frontal gyrus; SMA, supplementary motor area; STG, superior temporal gyrus; pSTG, posterior STG. L, left; R, right.

A 2 (priming type: ASP, ANSP) × 2 (perceived laterality relationship: separation, co-location) repeated-measured ANOVA was conducted, and no suprathreshold cluster was activated by the interaction. We marked out clusters activated by the ASP effect (i.e., “ASP > ANSP”) from these by the main effect of priming type, using a voxel-wise threshold of *p* < 0.001, uncorrected (clusters activated by the main effect of priming type are in both warm and cold colors in Figure 3A). The ASP effect activated the left motor cortex, left IFG, left posterior superior temporal gyrus, left inferior parietal region, bilateral putamen, and the right cerebellum (as shown in warm color clusters in Figure 3A). Meanwhile, we marked out clusters activated by the spatial unmasking effect (i.e., “perceived separation > perceived co-location”) from these by the main effect of perceived laterality relationship, using a voxel-wise threshold of *p* < 0.001, uncorrected (clusters activated by the main effect of perceived laterality relationship are in both warm and cold colors in Figure 3B). The spatial unmasking effect activated the bilateral precuneus, bilateral IFG, bilateral middle temporal gyrus, bilateral superior frontal gyrus extending into middle frontal gyrus (MFG), bilateral inferior parietal region, bilateral superior temporal gyrus, and bilateral putamen (as shown in warm color clusters in Figure 3B). The detailed results are also shown in Supplementary Table 2.

**FIGURE 4:**
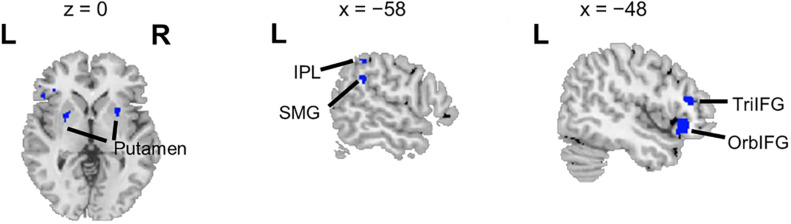
Overlapping brain areas activated by both the ASP effect and the spatial unmasking effect. L, left; R, right. Negative values of the *x* axis (the coronal axis) denote the left hemisphere. The variable *z* denotes the vertical axis.

Overlapping brain areas activated by both the ASP effect and the spatial unmasking effect were the bilateral putamen, left inferior parietal lobule (IPL), left supramarginal gyrus (SMG), left pars orbitalis of the IFG (OrbIFG), and left TriIFG (Figure 4), revealing shared neural bases of the unmasking effects of ASP and perceived separation.

### Correlations Between Brain Activation and Behavioral Improvement Due to ASP and Perceived Spatial Separation

**FIGURE 5:**
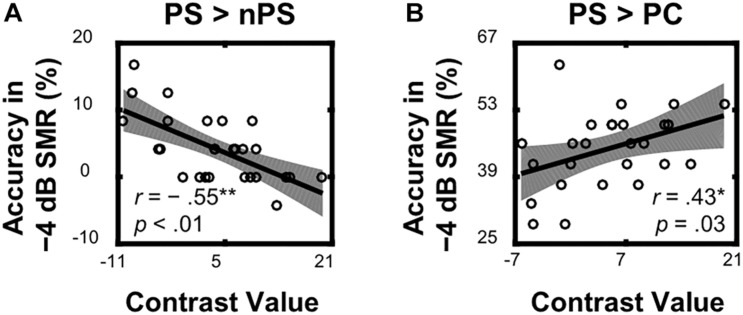
Correlations between neural activities of the overlapping left TriIFG and target recognition accuracy under a −4 dB SMR in behavioral testing. **(A)** Contrast values of the ASP effect under the perceived separation condition (“PS > nPS” contrast) were correlated with the corresponding contrasts of recognition accuracy. **(B)** Contrast values of the spatial unmasking effect under the ASP condition (“PS > PC” contrast) were correlated with the corresponding contrasts of recognition accuracy. The shades represent 95% confidence intervals.

Within the left TriIFG, the overlapping brain area activated by both the ASP effect and the spatial unmasking effect, the Spearman correlation analyses showed that contrast values for the ASP effect under the perceived separation condition (i.e., “PS > nPS” contrast) were significantly correlated with behavioral improvements for the corresponding contrast (*r* = −0.55, *p* < 0.01, Figure 5A), and contrast values for the spatial unmasking effect under the ASP condition (i.e., “PS > PC” contrasts) were also significantly correlated with behavioral improvements for the corresponding contrast (PS > PC: *r* = 0.43, *p* = 0.03, Figure 5B). The sizes of these correlations were moderate. No significant correlation was observed for the ASP effect under the perceived co-location condition (i.e., “PC > nPC” contrast) or the spatial unmasking effect under the ANSP condition (i.e., “nPS > nPC” contrast).

### Enhanced Functional Connectivity Due to ASP and Perceived Spatial Separation

**FIGURE 6:**
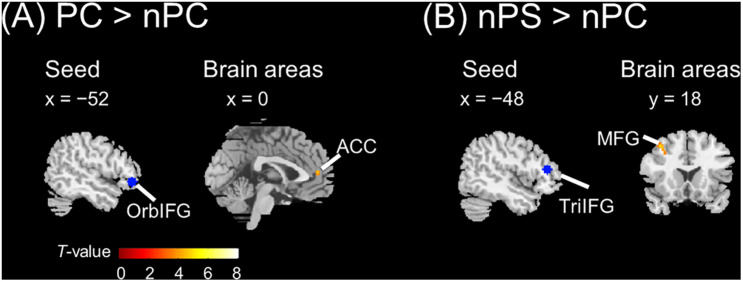
Brain areas exhibiting significant functional connectivity with the left IFG by the ASP effect and spatial unmasking effect. **(A)** The ASP effect under the perceived co-location condition (“PC > nPC” contrast) enhanced the functional connectivity between the left OrbIFG and the bilateral ACC. **(B)** The spatial unmasking effect under the ANSP condition (“nPS > nPC” contrast) enhanced the functional connectivity between the left TriIFG and the left MFG. All peaks survived an uncorrected threshold of *p* < 0.001 at the voxel level (*t* > 3.43), and each cluster consisted of more than 20 contiguous voxels. The positive value of *x* (the coronal axis) denotes the right hemisphere. Negative values of the *x* axis (the coronal axis) denote the left hemisphere. Positive values of *y* (the sagittal axis) denote the front. The blue areas are seed regions.

The seed regions were located in the left IFG. As shown in Figure 6A and Table 1, the ASP effect under the perceived co-location condition (i.e., “PC > nPC” contrast) enhanced the functional connectivity of the left OrbIFG [the seed locus at (−52, 38, −6)] with the bilateral anterior cingulate cortex (ACC). Meanwhile, the spatial unmasking effect under the ANSP condition (i.e., “nPS > nPC” contrast) enhanced the functional connectivity of the left TriIFG [the seed locus at (−48, 36, 14)] with the left MFG (Figure 6B and Table 1). Clusters that survived an uncorrected threshold of *p* < 0.001 at the voxel level with more than 20 contiguous voxels are reported.

**TABLE 1:** Areas of the brain exhibiting significant functional connectivity associated with the ASP effect under the perceived co-location condition (“PC > nPC” contrast) and with the spatial unmasking effect under the ANSP condition (“nPS > nPC” contrast).

Contrast	Seed	MNI coordinates (mm)			Statistics				Location
		*x*	*y*	*z*	*k*	*t*	*z* value	*p*_*unc*_	
PC > nPC	L. OrbIFG	−2	48	10	26	5.24	4.29	9.02E−06	L. ACC
nPS > nPC	L. TriIFG	−38	18	48	66	4.98	4.14	1.77E−05	L. MFG
		−34	20	38		4.05	3.53	2.07E−04	L. MFG

*The activation reported here survived an uncorrected threshold of *p* < 0.001 at the voxel level (*t* > 3.43), and the clusters consisted of more than 20 contiguous voxels. The MNI coordinates, *k* (the number of voxels), *t* value, *z* score, and uncorrected *p* values are provided. L, left.*

## Discussion

In the present study, we introduced ASP and perceived spatial separation to investigate how they improve recognition of the target speech in a “cocktail party” environment. The behavioral results showed that the benefits of ASP and perceived separation for speech recognition cannot be added. Neuroimaging results showed that neural underpinnings underlying the unmasking effects of ASP and perceived separation partly overlapped in brain areas related to the speech motor system, especially in the left IFG. These findings suggest the intersection of neural bases underlying two unmasking effects.

The behavioral results were consistent with previous findings that recognizing the target speech masked by two-talker speech can be improved by the prior knowledge of an early segment of the target speech and the perceived spatial separation (Freyman et al., 1999, 2004; Li et al., 2004; Wu et al., 2005, 2012, 2017a; Yang et al., 2007; Ezzatian et al., 2011; Zheng et al., 2016). Moreover, even though ASP improved the speech recognition in two perceived laterality relationships under a moderate degree of masking (such as the −4 dB in this study), the benefit of ASP was more significant when the maskers were perceived to be co-located with the target than when they were perceived to be separated from the target (Freyman et al., 2004). That is, we observed the non-additive benefits of ASP and perceived separation to speech recognition. Considering the additive effect of two cues suggesting independent processing of them (Du et al., 2011; Bronkhorst, 2015; Lu et al., 2018), the behavioral results suggest intersecting processing of ASP and perceived spatial separation.

The above inference is supported by remarkable neuroimaging results, which showed overlaps in neural underpinnings underlying the unmasking effects of ASP and perceived spatial separation. These intersectant brain areas included bilateral putamen, left IPL, left SMG, left OrbIFG, and left TriIFG. The common feature of these brain substrates is their involvement in the speech motor representation (for putamen, Abutalebi et al., 2013; for SMG, Deschamps et al., 2014; for IPL, Hickok and Poeppel, 2000; Shum et al., 2011; for IFG, Klaus and Hartwigsen, 2019), indicating a notable and common role of the speech motor representation for different unmasking effects in a noisy environment. This finding is in accordance with the idea that the auditory-to-motor transformation is important for speech perception in challenging listening situations (Price, 2010; D’Ausilio et al., 2012; Wu et al., 2014) and suggests that the enhancement of the speech motor representation is possibly the common neural mechanism underlying both the unmasking effects of ASP and perceived separation in a multitalker condition. Specifically, the inferior parietal region, containing the IPL and the SMG, is an important component of the network for the sensorimotor integration of speech. The sensorimotor integration is likely to be an emulation process for speech perception in challenging scenarios (Nuttall et al., 2016). It is assumed that covert emulation is processed in parallel with external events through the generation of top-down predictions of ongoing listening events, and perceptual processing is modulated by the feedback generated by these predictions (Wilson and Knoblich, 2005; Hickok et al., 2011; Nuttall et al., 2016). Interestingly, previous studies have discovered mirror neurons in the IPL and the left IFG, even though these studies focused on how actions are processed (Fogassi et al., 2005; Chong et al., 2008; Kilner et al., 2009). In consideration of the roles of IPL and IFG in this study, it can be proposed that the assumed perceptual emulator beneficial for challenging speech perception (Wilson and Knoblich, 2005) may be also in these two areas. In short, the involvement of inferior parietal region (i. e., the IPL and the SMG) in both the unmasking effects of ASP and perceived separation suggests that the two unmasking cues might improve recognition of the target speech by promoting the sensorimotor integration of the target in a noisy environment.

This study also underlines the notable role of the left IFG in the unmasking effects of ASP and perceived spatial separation. The left IFG is critically involved in the speech motor representation, typically the speech production (Liakakis et al., 2011; Du et al., 2014; Klaus and Hartwigsen, 2019). Previous studies suggest that the left IPL contributes to the phonological storage mechanism, while the left frontal network supports subvocal rehearsal and articulatory representation (Hickok and Poeppel, 2000; Hickok et al., 2011). In the present study, the left IFG was not only activated by both unmasking effects, but was also significantly correlated with behavioral improvements caused by them. These results suggest that the improvement of speech recognition due to ASP and perceived separation may be substantially in charge of enhanced subvocal rehearsal and covert speech production. These processes might be especially beneficial to improve speech intelligibility under adverse listening conditions. Apart from this, both ASP and perceived separation enhanced the functional connectivity of the left IFG with brain areas related to the suppression of distractive speech signals (i. e., the ACC and the MFG, respectively; for the ACC, Botvinick et al., 2004; Orr and Weissman, 2009; Kim et al., 2013; for the MFG, Bledowski et al., 2004). Even though the seeds were not identical, both the left OrbIFG and the left TriIFG receive inputs from temporal cortex to support the semantic processing of speech (Friederici, 2012). The results indicate that both ASP and perceived separation can facilitate the collaboration of the semantic processing to the target speech and the suppression to non-target speech. Previous studies have reported that the left IFG plays important roles in the high-level processing of speech, including recovering words and meaning from an impoverished speech signal (Wild et al., 2012b), unifying speech information (Hagoort, 2005; Acheson and Hagoort, 2013), and semantic selection in semantic competition situations (Grindrod et al., 2008; Whitney et al., 2011, 2012). Accordingly, when dealing with complex scenes, the left IFG may be an integration node for high-level processes, such as selecting features of the target speech from competing information, binding features related to the target speech into a consolidated target object, and allocating selective attention to the target speech. It is supposed that when enhancing certain features related to the target speech (e. g., spatial location, the voice, and the content of speech sounds), the overall salience of the target may increase among non-targets. The specific attentional mechanisms and binding processing should be examined in the future work.

In addition to overlapping brain areas, ASP and perceived spatial separation activated brain areas specific to themselves. The results showed that the ASP effect activated motor cortices, including the left precentral gyrus, left postcentral gyrus, and left supplementary area. These areas are involved in the higher-order speech motor representation, such as articulatory planning and execution and predictive speech motor control (Pulvermüller et al., 2006; Connie et al., 2016; Hertrich et al., 2016). The spatial unmasking effect activated the MFG and the precuneus, which have also been reported in Zheng et al. (2016). Both the MFG and the precuneus are involved in the spatial processing, such as spatial attention control and spatial computations in a complex listening environment (for the MFG, Giesbrecht et al., 2003; for the precuneus, Shomstein and Yantis, 2006; Wu et al., 2007; Krumbholz et al., 2009; Zündorf et al., 2013). Furthermore, the spatial unmasking effect activated the inferior and middle temporal gyri responsible for sound-to-meaning representation (Buckner et al., 2000; Hickok and Poeppel, 2007; Simanova et al., 2014). Thus, ASP and perceived separation were also processed by their cue-specific brain substrates. Specifically, the ASP featured its neural underpinnings in the motor cortex, whereas the perceived separation involved dual pathways in the auditory system. Besides, their individualities may also reflect in the suppression of distractive speech signals. The ASP enhanced the functional connectivity of the ACC with the left IFG, but its main effect did not involve the ACC. While the perceived separation enhanced the functional connectivity of the MFG with the left IFG, its main effect also activated the MFG. Considering the ACC is involved in monitoring conflicts between targets and distractors (Botvinick et al., 2004), and the MFG is involved in the distractor processing (Bledowski et al., 2004), these results may indicate that the ASP enhances the suppression of distractors by allocating cognitive resources between targets and distractors. By contrast, along with this process, the perceived separation may also directly crack down on the distractor processing. Future work in this research area should explore the relationships between the commonality and the individuality of these unmasking effects. They likely work together to improve speech recognition under a complex listening environment.

In conclusion, our study introduced ASP and perceived spatial separation in a multitalker acoustic environment to investigate common neural bases underlying their unmasking effects. We observed that the benefits of perceived separation on speech recognition could not add to that of ASP, and the speech motor system, especially the left IFG, was responsible for both the unmasking effects of ASP and perceived separation. These findings suggest that the unmasking effects of ASP and perceived separation are not independently processed, and there exist common neural bases supporting both the unmasking effects of ASP and perceived separation.

## Data Availability Statement

The datasets presented in this article are not readily available because the data were identifiable and not anonymous for each participants. Requests to access the datasets should be directed to LLi, liangli@pku.edu.cn.

## Ethics Statement

The studies involving human participants were reviewed and approved by the Committee for Protecting Human and Animal Subjects at the School of Psychological and Cognitive Sciences at Peking University. The participants provided their written informed consent to participate in this study.

## Author Contributions

JW: conceptualization, formal analysis, investigation, methodology, software, validation, visualization, writing—original draft, and writing—review and editing. JC: funding acquisition, writing—original draft, and writing—review and editing. XY: methodology. LLiu: formal analysis. CW: formal analysis, methodology, and software. LLu: methodology. LLi: conceptualization, funding acquisition, methodology, project administration, resources, validation, writing—review and editing, and supervision. YW: supervision. All authors contributed to the article and approved the submitted version.

## Conflict of Interest

The authors declare that the research was conducted in the absence of any commercial or financial relationships that could be construed as a potential conflict of interest.

## Footnotes

1 http://www.fil.ion.ucl.ac.uk/spm/

2 http://marsbar.sourceforge.net/

3 http://www.nitrc.org/projects/gppi/

## Abbreviations

ACC: anterior cingulate cortex
ANSP: auditory non-speech priming
ASP: auditory speech priming
ASSN: amplitude-modulated speech-spectrum noise
fMRI: functional magnetic resonance imaging
IFG: inferior frontal gyrus
IPL: inferior parietal lobule
MFG: middle frontal gyrus
nPS: ANSP and separation condition
nPC: ANSP and co-location condition
OrbIFG: pars orbitalis of IFG
PC: ASP and co-location condition
PS: ASP and separation condition
PPI: psychophysiological interaction
SMG: supramarginal gyrus
SMR: signal-to-masker ratio
TriIFG: par triangularis of IFG.
