# Supplementary material for: Common Brain Substrates Underlying Auditory Speech Priming and Perceived Spatial Separation
Source: Front Neurosci. 2021 Jun 17;15:664985. doi: 10.3389/fnins.2021.664985 (PMC8247760; doi:10.3389/fnins.2021.664985)
Supplement: Supplementary file 1 [file Table_1.DOCX]

Supplementary Material

Supplementary Table 1. *Recognition accuracy (%) of target speech under different priming types, perceived laterality relationships, and SMRs*

| SMR (dB) | Perceived Spatial Separation | | | |  | Perceived Spatial Co-location | | | |
| --- | --- | --- | --- | --- | --- | --- | --- | --- | --- |
|  | ASP | ANSP | *F* | *p* |  | ASP | ANSP | *F* | *p* |
| 0 | 90.74 (5.90) | 98.96 (2.31) | 69.06^***^ | < .001 |  | 86.92 (8.04) | 84.49 (11.38) | 2.43 | .128 |
| -4 | 97.22 (2.64) | 92.59 (5.19) | 29.05^***^ | < .001 |  | 51.51 (9.14) | 41.09 (10.26) | 27.44^***^ | < .001 |
| -8 | 92.25 (6.77) | 92.36 (5.77) | .01 | .919 |  | 30.32 (11.07) | 11.34 (8.78) | 86.91^***^ | < .001 |
| -12 | 79.17 (7.72) | 84.84 (8.22) | 13.24^***^ | < .001 |  | 12.50 (6.14) | 7.99 (7.21) | 11.67^**^ | .002 |

Note, ** *p* < .01, *** *p* < .001, *df1* = 1, *df2* = 35
