# Supplementary material for: Common Brain Substrates Underlying Auditory Speech Priming and Perceived Spatial Separation
Source: Front Neurosci. 2021 Jun 17;15:664985. doi: 10.3389/fnins.2021.664985 (PMC8247760; doi:10.3389/fnins.2021.664985)
Supplement: Supplementary file 2 [file Table_2.DOCX]

Supplementary Material

Supplementary Table 2. *Regions of the brain activated by the main effects of priming type and of perceived laterality relationship*

| Contrast | MNI Coordinates  (mm) | | |  | Statistics | | | | Location | Post-hoc |
| --- | --- | --- | --- | --- | --- | --- | --- | --- | --- | --- |
|  | x | y | z |  | k | *F* | *z*-value | *p*_unc_ |  |  |
| The Main Effect of Priming Type | **−52** | **−6** | **44** |  | **431** | **48.74** | **6.02** | **8.54E−10** | **L. PoCG** | **ASP > ANSP** |
|  | −44 | −12 | 44 |  |  | 37.25 | 5.38 | 3.78E−08 | L. PoCG | ASP > ANSP |
|  | −40 | −4 | 64 |  |  | 15.86 | 3.61 | 1.52E−04 | L. PrCG | ASP > ANSP |
|  | **−58** | **0** | **20** |  | **1765** | **48.16** | **5.99** | **1.02E−09** | **L. PoCG** | **ASP > ANSP** |
|  | −52 | 38 | −6 |  |  | 42.19 | 5.67 | 7.06E−09 | L. OrbIFG | ASP > ANSP |
|  | −52 | 12 | 10 |  |  | 35.63 | 5.27 | 6.65E−08 | L. OperIFG | ASP > ANSP |
|  | **26** | **20** | **56** |  | **224** | **32.54** | **5.07** | **2.01E−07** | **R. SFG** | **ANSP > ASP** |
|  | 28 | 8 | 48 |  |  | 26.57 | 4.62 | 1.87E−06 | R. MFG | ANSP > ASP |
|  | 34 | 14 | 40 |  |  | 18.81 | 3.93 | 4.27E−05 | R. MFG | ANSP > ASP |
|  | **−4** | **−2** | **58** |  | **149** | **32.51** | **5.07** | **2.03E−07** | **L. SMA** | **ASP > ANSP** |
|  | **6** | **−56** | **44** |  | **810** | **31.07** | **4.97** | **3.43E−07** | **R. Precuneus** | **ANSP > ASP** |
|  | −14 | −48 | 40 |  |  | 26.79 | 4.64 | 1.72E−06 | L. Precuneus | ANSP > ASP |
|  | −4 | −50 | 42 |  |  | 26.28 | 4.60 | 2.09E−06 | L. Precuneus | ANSP > ASP |
|  | **−56** | **−44** | **22** |  | **967** | **29.61** | **4.86** | **5.90E−07** | **L. STG** | **ASP > ANSP** |
|  | −54 | −42 | 30 |  |  | 27.48 | 4.70 | 1.32E−06 | L. SMG | ASP > ANSP |
|  | −54 | −50 | 46 |  |  | 25.47 | 4.54 | 2.87E−06 | L. IPL | ASP > ANSP |
|  | **26** | **6** | **−6** |  | **124** | **29.32** | **4.84** | **6.57E−07** | **R. Putamen** | **ASP > ANSP** |
|  | 30 | −8 | −8 |  |  | 27.76 | 4.72 | 1.19E−06 | R. Putamen | ASP > ANSP |
|  | 26 | 2 | 6 |  |  | 24.62 | 4.47 | 4.00E−06 | R. Putamen | ASP > ANSP |
|  | **22** | **−70** | **−50** |  | **274** | **28.10** | **4.75** | **1.04E−06** | **R. Cerebelum8** | **ASP > ANSP** |
|  | 20 | −74 | −40 |  |  | 25.11 | 4.51 | 3.30E−06 | R. Cerebelum. Crus2 | ASP > ANSP |
|  | 12 | −78 | −32 |  |  | 22.60 | 4.29 | 8.94E−06 | R. Cerebelum. Crus2 | ASP > ANSP |
|  | **−26** | **4** | **2** |  | **146** | **27.70** | **4.71** | **1.21E−06** | **L. Putamen** | **ASP > ANSP** |
|  | −24 | 12 | 2 |  |  | 24.02 | 4.41 | 5.08E−06 | L. Putamen | ASP > ANSP |
|  | −20 | 6 | 10 |  |  | 20.62 | 4.11 | 2.01E−05 | L. Putamen | ASP > ANSP |
|  | **52** | **0** | **−14** |  | **116** | **27.23** | **4.68** | **1.45E−06** | **R. STG** | **ANSP > ASP** |
|  | **46** | **−74** | **36** |  | **115** | **26.95** | **4.66** | **1.61E−06** | **R. Angular gyrus** | **ANSP > ASP** |
|  | 46 | −64 | 26 |  |  | 11.76 | 3.10 | 9.70E−04 | R. MOG | ANSP > ASP |
|  | **44** | **−58** | **18** |  | **214** | **22.14** | **4.25** | **1.08E−05** | **R. MTG** | **ANSP > ASP** |
|  | 46 | −50 | 18 |  |  | 21.06 | 4.15 | 1.67E−05 | R. MTG | ANSP > ASP |
|  | 54 | −54 | 12 |  |  | 16.19 | 3.65 | 1.32E−04 | R. MTG | ANSP > ASP |
|  | **4** | **60** | **20** |  | **368** | **20.60** | **4.10** | **2.03E−05** | **R. medial SFG** | **ANSP > ASP** |
|  | 6 | 60 | 10 |  |  | 18.97 | 3.94 | 4.00E−05 | R. medial SFG | ANSP > ASP |
|  | −6 | 62 | 14 |  |  | 18.39 | 3.89 | 5.12E−05 | L. medial SFG | ANSP > ASP |
|  | **−42** | **−70** | **22** |  | **136** | **19.13** | **3.96** | **3.74E−05** | **L. MOG** | ANSP > ASP |
|  | −48 | −74 | 16 |  |  | 17.04 | 3.74 | 9.11E−05 | L. MOG | ANSP > ASP |
| The Main Effect of Perceived Laterality Relationship | **6** | **−54** | **56** |  | **2667** | **131.31** | **Inf.** | **4.44E−16** | **R. Precuneus** | **Sepa. > Colo.** |
|  | −6 | −54 | 58 |  |  | 82.95 | 7.40 | 6.68E−14 | L. Precuneus | Sepa. > Colo. |
|  | −6 | −52 | 48 |  |  | 60.00 | 6.55 | 2.92E−11 | L. Precuneus | Sepa. > Colo. |
|  | **50** | **−24** | **10** |  | **2096** | **103.32** | **Inf.** | **7.77E−16** | **R. STG** | **Sepa. > Colo.** |
|  | 54 | −26 | 26 |  |  | 38.58 | 5.46 | 2.38E−08 | R. SMG | Sepa. > Colo. |
|  | 42 | −64 | 16 |  |  | 31.93 | 5.03 | 2.50E−07 | R. MTG | Sepa. > Colo. |
|  | **−44** | **−14** | **−28** |  | **2288** | **62.24** | **6.64** | **1.54E−11** | **L. ITG** | **Sepa. > Colo.** |
|  | −48 | −34 | −22 |  |  | 56.31 | 6.39 | 8.54E−11 | L. ITG | Sepa. > Colo. |
|  | −66 | −52 | −2 |  |  | 56.17 | 6.38 | 8.89E−11 | L. MTG | Sepa. > Colo. |
|  | **−34** | **30** | **−16** |  | **1487** | **60.28** | **6.56** | **2.69E−11** | **L. OrbIFG** | **Sepa. > Colo.** |
|  | −50 | 28 | −2 |  |  | 42.44 | 5.69 | 6.51E−09 | L. TriIFG | Sepa. > Colo. |
|  | −48 | 36 | 14 |  |  | 34.75 | 5.22 | 9.08E−08 | L. TriIFG | Sepa. > Colo. |
|  | **26** | **6** | **−6** |  | **400** | **56.82** | **6.41** | **7.34E−11** | **R. Putamen** | **Sepa. > Colo.** |
|  | 30 | −6 | −4 |  |  | 40.14 | 5.55 | 1.41E−08 | R. Putamen | Sepa. > Colo. |
|  | 32 | −16 | 0 |  |  | 37.41 | 5.39 | 3.56E−08 | R. Putamen | Sepa. > Colo. |
|  | **−46** | **−28** | **6** |  | **778** | **51.77** | **6.17** | **3.34E−10** | **L. STG** | **Sepa. > Colo.** |
|  | −64 | −32 | 20 |  |  | 31.82 | 5.02 | 2.61E−07 | L. STG | Sepa. > Colo. |
|  | −62 | −38 | 32 |  |  | 29.70 | 4.87 | 5.71E−07 | L. SMG | Sepa. > Colo. |
|  | **2** | **16** | **50** |  | **761** | **40.17** | **5.55** | **1.39E−08** | **L. SMA** | **Colo. > Sepa.** |
|  | 4 | 12 | 60 |  |  | 38.39 | 5.45 | 2.55E−08 | R. SMA | Colo. > Sepa. |
|  | 6 | 22 | 42 |  |  | 33.42 | 5.13 | 1.46E−07 | R. medial SFG | Colo. > Sepa. |
|  | **32** | **32** | **−16** |  | **624** | **40.03** | **5.55** | **1.46E−08** | **R. OrbIFG** | **Sepa. > Colo.** |
|  | 52 | −10 | −24 |  |  | 31.41 | 4.99 | 3.03E−07 | R. MTG | Sepa. > Colo. |
|  | 46 | 16 | −20 |  |  | 23.60 | 4.38 | 6.01E−06 | R. pole of STG | Sepa. > Colo. |
|  | **50** | **18** | **8** |  | **386** | **36.62** | **5.34** | **4.69E−08** | **R. OperIFG** | **Colo. > Sepa.** |
|  | 38 | 18 | 8 |  |  | 35.48 | 5.26 | 7.01E−08 | R. Insula | Colo. > Sepa. |
|  | 30 | 26 | 4 |  |  | 32.63 | 5.07 | 1.94E−07 | R. Insula | Colo. > Sepa. |
|  | **−24** | **8** | **−10** |  | **167** | **34.59** | **5.21** | **9.61E−08** | **L. Putamen** | **Sepa. > Colo.** |
|  | −28 | 4 | −2 |  |  | 28.99 | 4.81 | 7.45E−07 | L. Putamen | Sepa. > Colo. |
|  | −32 | −10 | −4 |  |  | 24.86 | 4.49 | 3.64E−06 | L. Putamen | Sepa. > Colo. |
|  | **36** | **−28** | **−20** |  | **226** | **33.82** | **5.16** | **1.26E−07** | **R. fusiform gyrus** | **Sepa. > Colo.** |
|  | 30 | −44 | −6 |  |  | 29.24 | 4.83 | 6.77E−07 | R. PHG | Sepa. > Colo. |
|  | 24 | −32 | −14 |  |  | 19.57 | 4.01 | 3.10E−05 | R. PHG | Sepa. > Colo. |
|  | **−18** | **34** | **46** |  | **561** | **32.41** | **5.06** | **2.11E−07** | **L. SFG** | **Sepa. > Colo.** |
|  | −26 | 34 | 34 |  |  | 28.42 | 4.77 | 9.24E−07 | L. SFG | Sepa. > Colo. |
|  | −24 | 22 | 50 |  |  | 24.64 | 4.47 | 3.97E−06 | L. MFG | Sepa. > Colo. |
|  | **14** | **44** | **44** |  | **141** | **23.20** | **4.34** | **7.02E−06** | **R. medial SFG** | **Sepa. > Colo.** |
|  | 18 | 36 | 42 |  |  | 16.49 | 3.68 | 1.15E−04 | R. SFG | Sepa. > Colo. |
|  | **4** | **46** | **−14** |  | **120** | **20.85** | **4.13** | **1.83E−05** | **R.** **orbital MFG** | **Sepa. > Colo.** |
|  | −4 | 56 | −12 |  |  | 15.35 | 3.55 | 1.91E−04 | L. orbital MFG | Sepa. > Colo. |

The activation reported here survived the cluster-level correction for multiple comparisons (*P*_FWE_ < .05 at cluster level, *F* > 11.69), consequently, the clusters that were activated by the main effect of priming type were more than 115 contiguous voxels and the clusters that were activated by the main effect of perceived laterality relationship were more than 120 contiguous voxels. The MNI Coordinates, k (the number of voxels), *F*-value, *z*-score, and uncorrected *p*-values are provided. Additionally, clusters are labelled to distinguish the contributions of specific contrasts to main effects by post-hoc *t*-tests. IPL, inferior parietal lobule; ITG, inferior temporal gyrus; MFG, middle frontal gyrus; MOG, middle occipital gyrus; MTG, middle temporal gyrus; OperIFG, pars operculum of inferior frontal gyrus; OrbIFG, pars orbitalis inferior frontal gyrus; PHG, parahippocampal gyrus; PoCG, postcentral gyrus; PrCG, precentral gyrus; SFG, superior frontal gyrus; SMA, supplemental motor area; SMG, supramarginal gyrus; STG, superior temporal gyrus; TriIFG, par triangularis inferior frontal gyrus; L, left; R, right. ANSP, auditory non-speech priming; ASP, auditory speech priming; Colo, co-location; Sepa, separation. The bold numbers and words highlight the maximum peak of these clusters.
